# Association genetics of acetophenone defence against spruce budworm in mature white spruce

**DOI:** 10.1186/s12870-018-1434-y

**Published:** 2018-10-12

**Authors:** Mebarek Lamara, Geneviève J. Parent, Isabelle Giguère, Jean Beaulieu, Jean Bousquet, John J. MacKay

**Affiliations:** 1 Forest Research Centre and Institute for Systems and Integrative Biology, Département des sciences du bois et de la forêt, Université Laval, Qc, Québec, G1V 0A6 Canada; 2 Department of Plant Sciences, University of Oxford, Oxford, OX1 3RB UK; 3 Canada Research Chair in Forest Genomics, Université Laval, Qc, Québec, G1V 0A6 Canada

**Keywords:** Association genetics, Phenolic compounds, *Pgβglu-1* expression, Spruce budworm, White spruce, Metabolic trade-offs

## Abstract

**Background:**

Outbreaks of spruce budworm (SBW, *Choristoneura fumiferana* Clem.) cause major recurrent damage in boreal conifers such as white spruce **(***Picea glauca* [Moench] Voss) and large losses of forest biomass in North America. Although defensive phenolic compounds have recently been linked to chemical resistance against SBW, their genetic basis remains poorly understood in forest trees, especially in conifers. Here, we used diverse association genetics approaches to discover genes and their variants that may control the accumulation of acetophenones, and dissect the genetic architecture of these defence compounds against SBW in white spruce mature trees.

**Results:**

Out of 4747 single nucleotide polymorphisms (SNPs) from 2312 genes genotyped in a population of 211 unrelated individuals, genetic association analyses identified 35 SNPs in 33 different genes that were significantly associated with the defence traits by using single-locus, multi-locus and multi-trait approaches. The multi-locus approach was particularly effective at detecting SNP–trait associations that explained a large fraction of the phenotypic variance (from 20 to 43%). Significant genes were regulatory including the NAC transcription factor, or they were involved in carbohydrate metabolism, falling into the binding, catalytic or transporter activity functional classes. Most of them were highly expressed in foliage. Weak positive phenotypic correlations were observed between defence and growth traits, indicating little or no evidence of defence-growth trade-offs.

**Conclusions:**

This study provides new insights on the genetic architecture of tree defence traits, contributing to our understanding of the physiology of resistance mechanisms to biotic factors and providing a basis for the genetic improvement of the constitutive defence of white spruce against SBW.

**Electronic supplementary material:**

The online version of this article (10.1186/s12870-018-1434-y) contains supplementary material, which is available to authorized users.

## Background

Trees use a battery of constitutive and inducible defence strategies to limit the damage of herbivory from insects over their long life span [1, 2]. Constitutive chemical defence barriers are particularly well developed in conifers, which produce a wide range of secondary metabolites such as oleoresin terpenoids and phenolic compounds to reduce herbivore attacks [3–6]. The arsenal of constitutive and inducible terpenes that are produced by conifers such as spruce, pine or fir have become some of the best studied secondary metabolites in trees, particularly in regard to the mechanisms of synthesis and the molecular bases of their regulation [7–11]. However, the molecular basis of heritable variation in chemical defences is only partially understood.

The spruce budworm (SBW) *Choristoneura fumiferana* Clemens (Lepidoptera: Tortricidae) is one of the most destructive native insect pests in coniferous and mixed forests of North America, particularly in the East [12–15]. In the last decade, recurrent outbreaks of SBW in Canada have caused high levels of tree mortality in fir and spruce trees through intensive leaf herbivory [16]. The outbreaks have spread over millions of hectares [17] and caused losses varying from 3 to 68 m^3^/ha of wood [16] depending on stand and region, with substantial damages occurring in both natural and plantation forests [18]. SBW larvae preferentially feed on the new foliage of conifers, which include, in decreasing order of susceptibility, balsam fir (*Abies balsamea* [L.] Mill.), white spruce (*Picea glauca* [Moench] Voss), red spruce (*P. rubens* Sarg.) and black spruce (*P. mariana* [Mill.] BSP) [18–20]. Despite the ecological and economic importance of these spruce and fir trees in North American forests, little is known of naturally-occurring defence mechanisms against SBW.

Quantitative genetics studies of chemical defence compounds such as monoterpenes in conifers have reported considerable intraspecific variation at the phenotypic level and relatively high estimates of heritability, which indicates the strong genetic control underlying these traits [5, 21–23]. Variability in some phenolic compounds and other secondary metabolites that may accumulate in leaf tissues is also under genetic control in trees [24–26]. For instance, the accumulation of sideroxylonal was shown to be highly heritable in eucalypt species [24, 27]; however, compared to terpenes, the biosynthesis and genetic control of phenolic compounds involved in defence against insects is less well understood in conifers. The shikimic acid and phenylpropanoid pathways form the core biosynthetic route leading to the production of both defensive and structural phenolic compounds such as lignin [28], but the mechanisms by which the former may accumulate are largely unknown in conifers.

In white spruce trees, constitutive chemical defence against SBW has been linked to the accumulation of piceol and pungenol, which belong to a class of phenolic compounds known as acetophenones [19, 29]. Considerable variation was observed in this naturally-occurring resistance mechanism and it was shown to be linked to the expression of the *Pgβglu-1* gene [29]. Population [30], sib and clonal analyses, showed that variation in acetophenone concentrations was highly heritable and positively impacted white spruce fitness. Laboratory feeding experiments also showed a decrease in SBW larvae survival [19]. In contrast, the glycosylated conjugates picein and pungenin accumulated in both resistant and non-resistant trees and were not biologically active against SBW [19]. These findings have raised questions regarding the molecular basis of the genetic control underlying these defence traits and the considerable natural genetic variation observed at the population level.

Association genetics approaches are often used to dissect complex traits in forest trees including wood quality [31–34] and defence against insect herbivory [5, 6, 35]. Single–locus association studies have identified associations between SNPs in candidate genes and defence against insect herbivory in *Eucalyptus* [35] and *Pinus* [5, 6]. However, most of the marker-trait associations only explained a small proportion of the phenotypic variation. This is due to the fact that variation in complex traits appears to be based on many loci with small effects [36]. Alternative approaches may be more effective at uncovering the network of gene effects which underpins phenotypic variations. For example, multi-locus analyses have been developed for more effective capture of combined effects [37] and multi-trait models have also been used to account for trait interactions [38].

The high constitutive levels of acetophenones reported for white spruce [39] also raise questions regarding possible trade-offs between defence and growth. The production cost of phenolic compounds could be relatively high and may compete with the formation of new tissues or the accumulation of reserves. Different hypotheses related to the balance of energy between growth and defence have been proposed [40–43], but little evidence of trade-offs has been observed in trees to date. Such trade-offs would be detected as negative phenotypic correlations between defence and growth traits and could have consequences on breeding strategies for improved defence against SBW in white spruce [44].

This study pursued two major objectives: (1) to identify genes and SNPs associated with variation in acetophenone concentrations. To date, the level of acetophenone aglycons has been explained in part by *Pgβglu-1* expression but it accounted for less than half of the variation [29]. Moreover, variation in the acetophenone glucoside picein remains unexplained. We thus used three different association study approaches to identify genes associated with phenolic compounds and *Pgβglu-1* expression as quantitative defence traits. (2) to examine potential trade-offs between acetophenone defences and growth given that phenolic compounds such as picein accumulate to very high concentrations in the foliage of some trees but not in others.

## Methods

The four defence traits assessed in this study were the acetophenones piceol and pungenol, the expression levels of the *Pgβglu-1*gene, which is responsible for their release, and the acetophenone glucoside picein. These traits were first described in [19] and in [29]. Sampling and laboratory analyses are summarized in the following sections, but more details are available from [39]. In a separate analysis, the phenotypic data of three growth traits (tree height in m (Ht), stem diameter at breast height in cm (DBH), and growth ring width averaged from pith to bark in cm (RW)) from [45] were used to assess defence-growth trade-offs.

### Plant materials

Foliage of 211 unrelated 38-year-old mature white spruce (*Picea glauca*) trees, each from a distinct open-pollinated family and representing 42 geographic origins (provenances), were sampled in a provenance-progeny test established by the Canadian Forest Service in the field with three-year old trees in 1979 at the site of Mastigouche, Québec, Canada (46°38’N, 73°13’W) (described in [45]). Only current-year foliage was sampled from the north side of the mid-crown on 24 July 2014 (trees aged 38), frozen immediately in liquid nitrogen after removal from the trees and stored at − 80 °C. The foliage was ground to a fine powder using a MixerMill 300 (Retsch) and steel grinding balls cooled in nitrogen. Powdered tissue was stored at − 80 °C until further analyses.

The sampling was non-destructive and the trees were part of an experimental plantation established for research on land of the government of Québec. A collaborative research agreement between the organizations as part of the Arborea II project gave permission for the sampling, which followed guidelines of the institutions involved in the research and in force in Québec (Canada).

### RNA extraction and transcript determination assays

Total RNA was extracted as in [46] with modifications as in [47] and stored at − 80 °C. The total RNA concentration was determined using a NanoDrop 1000 (Thermo Fisher Scientific, Wilmington, DE, USA) and assessed for quality with an Agilent 2100 Bioanalyzer and RNA 6000 Nano Kit LabChips (Agilent Technologies Inc.). Only RNA isolates with an integrity score (RIN) of 7.0 or more were used for analyses. Reverse transcriptase-quantitative PCR (RT-qPCR) with gene-specific primers was used to quantify transcript accumulation levels of the *Pgβglu-1* gene (see [29] for more details).

### Acetophenone extractions and determinations

The hydroxyl-acetophenones piceol and pungenol and the hydroxyl-acetophenone glucoside picein were extracted as described in [39]. Assays were conducted on a LC (Agilent 1200 series) coupled to a MS detector (Agilent 6210 TOF). Acetophenones were separated in a pre-column Polaris MetaGuard 4.6 mm and a column Polaris 250 mm × 4.6 mm C18-A, particular size 5 μm (Agilent Technologies Inc.). The solvent and solvent gradient were as described in [39]. The column flow rate was 1.5 ml min^− 1^ and ten microlitres of extract were injected. Quantification was done using external calibration curves for picein, piceol and pungenol. No pungenin was commercially available.

### Genotypic data

High-quality genotyping data based on single nucleotide polymorphisms (SNPs) were obtained using an Infinium iSelect genotyping chip (Illumina, San Diego, CA) and were previously described [45]. In the current study, a cut-off of 0.10 for minor allele frequency (MAF) was used, resulting in a set of 4767 high-quality SNPs in 2312 genes without any missing genotypes from a starting dataset of 6385 SNPs in 2652 genes. The gene sequences are described in the white spruce gene catalogue [48] and genes were selected based on multiple criteria as described in [49] and the Supporting Information in [34]. Briefly, these criteria were related to 1) predicted functions relevant for wood formation, growth, phenology, and adaptation to biotic and abiotic factors as indicated by database searching and scientific literature from *Arabidopsis* and poplar (e.g. [50–52]; 2) expressional candidate genes related to phenology [53] and vascular tissue differentiation [47]; 3) overexpression of R2R3-MYB genes, HD-zips and other transcription factors in spruce trees [54–57]; 4) co-localization with QTLs for bud flush, bud set and height growth [58]; and 5) genes harbouring SNPs implicated in local adaptation [59]. The genes were well distributed across the 12 linkage groups of white spruce [60].

### Simulations

Simulations were used to assess the effectiveness of the multi-locus mixed model (MLMM) and single-locus mixed model (SLMM) in detecting associations under different genetic architectures of the complex traits in the present population. Simulated phenotypic data sets were generated by simulating genetic effects based on real genotype data (4767 SNPs) drawn from this study using the R-package BGLR [61]. A theoretical normally distributed phenotypic trait was simulated for the 211 trees under two different scenarios differing in the number of SNPs controlling the phenotype; scenario I, 10 SNPs and scenario II, 50 SNPs. For both scenarios, we tested three different heritability levels, i.e. 0.50, 0.75 and 1.

### Association analyses

Data for the four investigated defence traits were normalized using the rank-based inverse normal transformation, implemented as the *rntransform* function in the GenABEL R Library [62] in order to comply with assumptions of association genetics testing that residuals be normally distributed. Principal component analysis (PCA) and a pairwise kinship matrix were used to assess for the presence of population structure in the set of 211 trees using the 4767 SNPs. The association analyses between SNPs and traits were performed using the three following approaches.

The single-locus mixed model (SLMM) implemented in TASSEL v5.2.1 [63] as described by [64] was used to take into account potential relatedness among the 211 trees as well as a weak population structure previously noted [32] so to remove any spurious association effects. We set a uniform threshold *P* < 2 × 10^− 4^ (calculated according to *P* = 1/n; n = total number of SNPs used in the analysis), which is roughly equivalent to a Bonferonni correction [65, 66], to determine if the SNP markers were significantly associated with the four defence traits for the different analyses.

The modified version of multi-locus mixed model (MLMM), as developed by [37] where PCA scores and kinship coefficients are defined as cofactors, was used to further identify SNPs potentially associated with the four defence traits. The approach relies on a simple, stepwise mixed-model regression with forward inclusion and backward elimination while re-estimating the genetic and error variances at each step of the regression. This method may well lead to higher detection power and a lower FDR when compared with traditional single-locus approaches [37]. For each phenotype, the percentage of phenotypic variation explained (PVE) by markers was determined at the optimal step. The multi-trait mixed model (MTMM) [38] was used to analyse pairs of correlated traits. This approach is based on the principle that measurements taken for the correlated traits may be combined to increase the power to detect common SNPs in genetic association with both traits [38, 67, 68].

### Trade-offs between defence and growth traits

We investigated whether there may be trade-off relationships between the constitutive defence and three growth traits, tree height in m (Ht), stem diameter at breast height in cm (DBH), and growth ring width averaged from pith to bark in cm (RW) as reported previously in [45]. First, pairwise Pearson correlation coefficients were determined between all traits to estimate the magnitude of trade-offs using the transformed data. Second, a principal component analysis (PCA) was conducted using the *prcomp* function implemented in R [69] to graphically illustrate the relationship between SBW defence traits represented by acetophenone compounds and *Pgβglu-1* transcripts on one hand, and growth traits on the other hand by examining the biplot graphics. Third, association analyses were performed between SNPs and all of the growth and defence traits by using permissive statistical test conditions (SLMM method, threshold of *P* < 0.05 without correction for multiple testing [34]) in order to uniquely determine the extent of overlap among the sets of genes that may be linked to the different traits.

## Results

### Phenotypic variation

**Fig. 1:**
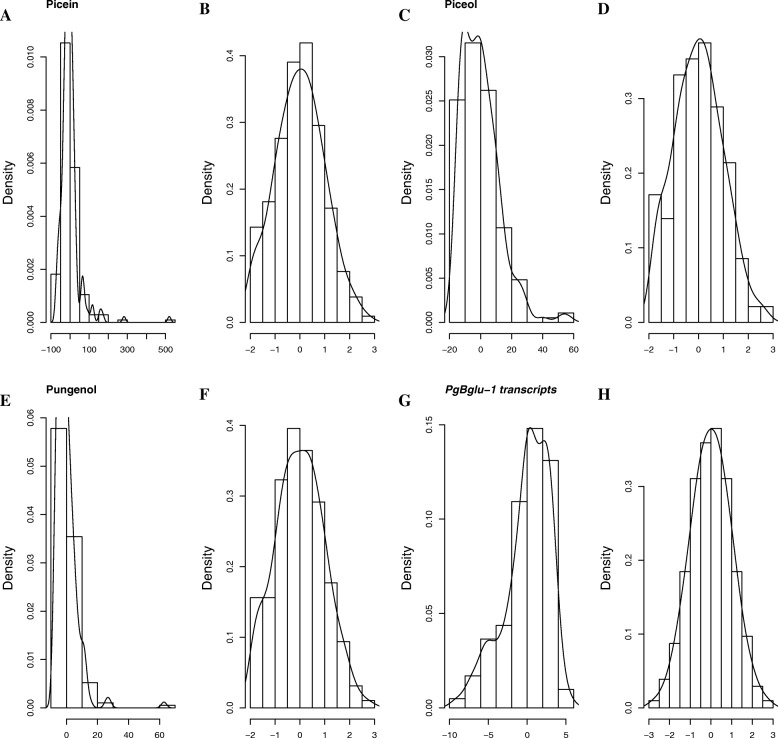
Histogram and density plot showing residual distribution in all traits. **a**, **c**, **e**, **g** before normalization and (**b**, **d**, **f**, **h**) after normalization

Table 1 shows the summary statistics for the four defence traits determined in 211 unrelated trees each representing a different open-pollinated family from 42 natural populations [45] . A broad range of variation was observed for each trait. In particular, acetophenone defence compounds accumulated to high levels in some trees and were undetected in others (Table 1); in addition, the data were not normally distributed and skewed toward low values (Fig. 1a, c, e). Similar observations were made for the *Pgβglu-1* transcript levels though the distribution was skewed toward high levels (Fig. 1g). Data were transformed using the rank-based inverse normal transformation such that residuals were normalized (Fig. 1b, d, f, h) prior to conducting the association genetics analyses that follow.

**Table 1:** Summary statistics of constitutive defence traits in the white spruce association population

Defence traits	Number of trees	Minimum	Maximum	Median	Mean
Picein (mg/g)	210	0	590.2	53.3	62.4
Piceol (mg/g)	211	0	71.7	11.2	12.9
Pungenol (mg/g)	211	0	70.0	5.3	6.4
*Pgβglu-1* transcripts (ng/RNA)	206	5	180,500	4197	11,320

### Simulations

We used genotyping data for 4767 high-quality SNPs from 2312 candidate genes [45] to search for and analyse SNP-trait associations potentially controlling the defence phenotypes against SBW. The simulations used to assess the potential to detect SNPs in the study population indicated that the multi-locus mixed model (MLMM) detected a larger number of SNP-trait associations compared to the single-locus mixed model (SLMM) (Table 2). The power to detect SNP-trait associations declined when SNPs controlling the trait increased from 10 to 50, especially at a moderate heritability level.

**Table 2:** Simulation results of detecting significant^a^ SNP-trait associations using SLMM and MLMM approaches

Association approaches^b^	10 SNPs			50 SNPs		
	Heritability			Heritability		
	0.50	0.75	1.0	0.50	0.75	1.0
SLMM	0	3	6	0	0	3
MLMM	6	8	10	1	3	8

^a^The significant threshold used was *P* < 2 × 10^− 4^

^b^SLMM, single-locus mixed model; and MLMM, multi-locus mixed model

### Identification of SNPs and genes associated with defence traits

In a first step, we used the SLMM and MLMM approaches and identified a total of 31 SNPs in 29 genes that were significantly associated with variation in at least one of the acetophenone compounds and *Pgβglu-1* transcripts levels at the Bonferroni-corrected statistical threshold (−log *P* > 3.68, α = 1) (Table 3). The SLMM method identified eight significant associations involving seven different SNP (Table 3). The proportion of the phenotypic variation explained (PVE) by all significant SNPs varied from as little as 2.3% for piceol to as high as 11.2% for picein (Table 3). In contrast, significant associations were obtained for 26 SNPs with MLMM. Three of the SNPs were associated with the glucosylated phenolic compound picein and explained 20% of phenotypic variation; two of them were also identified with the SLMM approach. The acetophenone piceol was significantly associated with nine SNPs with a PVE of 43%, and pungenol was significantly associated with six SNPs with a PVE of 27%. A total of eight SNPs were significantly associated with *Pgβglu-1* transcripts and explained 23% of phenotypic variation. Our results indicate that the analysis carried out with the MLMM method by using the same SNPs genotyped in the population was more effective for detecting significant SNPs compared to the traditional SLMM approach (Table 3) as intended by its developers [37]. In total, five SNPs representative of 5 distinct genes were detected by SLMM, and 23 SNPs representative of 23 distinct genes were detected by MLMM, with three SNPs representative of three distinct genes in common between SLMM and MLMM, thus resulting in a total of 31 distinct SNPs representative of 29 genes detected by the two methods.

**Table 3:** SNPs significantly associated with defence traits in white spruce using SLMM and MLMM approaches^a^, and their combined percentage of phenotypic variation explained (PVE)

Defence traits	SLMM		MLMM	
	Nb. of SNPs^b^	PVE (%)	Nb. of SNPs^b^	PVE (%)
Picein	2 (2)^c^	11.2	3 (3)	20
Piceol	2 (2)	2.3	9 (9)	43
Pungenol	2 (2)	4.0	6 (6)	27
*Pgβglu-1* transcripts	2 (2)	8.2	8 (8)	23
Total number of distinct SNPs	8 (7)	–	26 (26)	–

^a^SLMM, single-locus mixed model; MLMM, multi-locus mixed model

^b^Number of significant SNPs associated with the trait variation

^c^In parentheses, number of significant genes

**Fig. 2:**
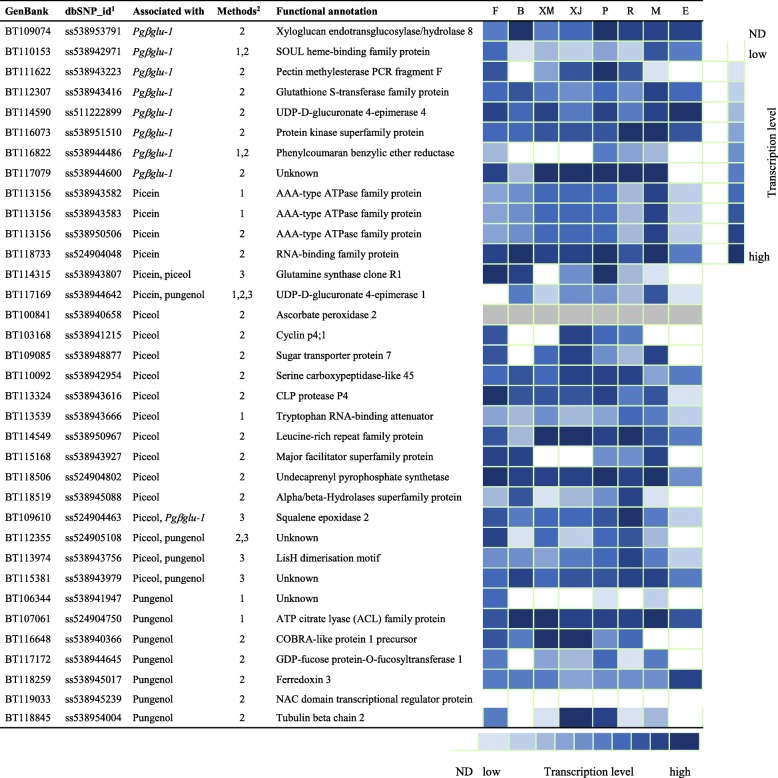
Heatmap of tissue-specific expression patterns of significantly associated genes and functional annotations. White spruce expression data are from the PiceaGenExpress database [70]. ^1^From [49]; ^2^Methods: 1, single-locus mixed model (SLMM); 2, multi-locus mixed model (MLMM), 3, multi-trait mixed model (MTMM). Columns represent vegetative tissues: F, foliage; B, vegetative buds; XM, xylem–mature; XJ, xylem–juvenile; P, phelloderm; R, adventitious roots; M, megagametophytes; E, embryogenic cells; transcript levels represent relative abundance classes within each tissue, grey is for missing data; ND, not detected

Piceol, pungenol and *Pgβglu-1* expression were previously reported to be moderately correlated [29]. Thus, the MTMM approach was used to search for significant SNPs in common between each pair of the traits used in this study. Three SNPs (from three distinct genes) were shared between piceol and pungenol, and four times one SNP (from distinct genes) were shared in other pairs of traits (Table 4). We thus identified a total of six significant SNPs (from as many distinct genes) associated with the combined traits, including four new SNPs identified only with the MTMM approach. The two other SNPs were also detected by using SLMM and/or MLMM approaches. In total using the three methods (SLMM, MLMM and MTMM) applied to the four defence traits, we identified 35 different SNPs representative of 33 distinct genes (Fig. 2).

**Table 4:** Number of significant SNPs associated with defence traits in white spruce using the multi-trait mixed model (MTMM) approach

Defence traits	Piceol	Pungenol	*Pgβglu-1* transcripts
Picein	1 (1)^a^	1 (1)	1 (1)
Piceol	–	3 (3)	1 (1)
Pungenol	–	–	0

^a^Number of significant genes in brackets

### Functional annotations and expression of genes associated with defence traits against spruce budworm

We began the characterization of the 33 genes containing the 35 SNPs significantly associated with the defence traits by conducting an analysis of the gene ontology (GO) terms associated to these functionally annotated genes. We found that the genes belonged essentially to three molecular functions: binding (GO:0005488; 6 genes), catalytic activity (GO:0003824; 19 genes) and transporter activity (GO:0005215; 2 genes). The catalytic activity category harboured the largest number of genes and involved several different enzymatic functions.

None of the genes were annotated as encoding enzymes of the shikimic or general phenylpropanoid pathways that are responsible for the synthesis of phenolic compounds used in the formation of acetophenones, despite the fact that most of the corresponding genes were represented on the SNP array (Additional file 1: Figure S1). In contrast, the four defence traits were associated with genes involved in carbohydrate metabolism, and they were annotated as xyloglucan endotransglucosylase/hydrolase 8 (XTH8), sugar transporter protein 7 (STP7), UDP-D-glucuronate 4-epimerase 1 (GAE1) and UDP-D-glucuronate 4-epimerase 4 (GAE4). Also, genes that bear regulatory functions, including the NAC transcriptional factor, suppressor of gamma response 1 (SOG1), and genes that are involved in response to different stimulus and stress, including ascorbate peroxidase (APX), glutathione S-transferases (GST) and phenylcoumaran benzylic ether reductase1 (PCBER1) were observed as carrying significant SNPs, as well as other genes of unknown functions.

Next, we examined gene expression profiles for the 33 genes identified to carry the 35 significant SNPs by using data from the PiceaGenExpress database comprised of microarray RNA profiles [70], which indicated variable expression across tissues. The expression data indicated that most of these genes were highly expressed in foliage and also expressed at variable levels in one or several other tissues (Fig. 2).

### Defence-growth trade-offs

**Fig. 3:**
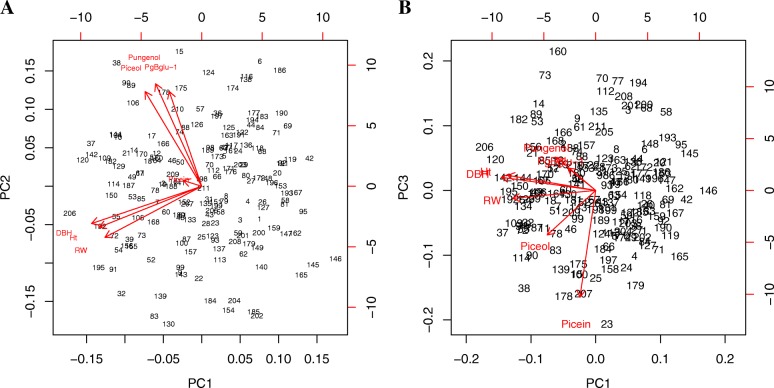
Biplots of the three first components of principal component analysis of defence and growth traits. **a**, principal component 2 (PC2) versus principal component 1 (PC1); **b**, principal component 3 (PC3) versus PC1. Abbreviations: RW, average ring width; Ht, tree height; DBH, stem diameter at breast height

No trade-offs were identified between levels of the key defensive compounds piceol and pungenol and growth traits. First, we calculated phenotypic correlations between three different growth traits, i.e. total tree height, stem diameter at breast height and growth ring width averaged from pith to bark [45]. Phenotypic correlations were generally low between piceol or pungenol and the three growth traits (Table 5). In fact, the largest coefficient of correlation (0.12) was observed between picein and average ring width and between piceol and stem diameter, which indicates no possible trade-off. Second, a PCA analysis was carried out considering all of the traits related to defence and growth and similar results were obtained (Fig. 3). The first principal component (PC1) explained 33% of the total variation (Fig. 3), while the second (PC2) and third one (PC3) explained 29% and 18% of the variation, respectively. PC1 was largely determined by growth traits, and variation of PC2 was controlled mostly by piceol, pungenol and the level of *Pgβglu-1* transcripts, whereas most of the variation of PC3 was controlled by picein (Table 6).

**Table 5:** Phenotypic correlations between defence traits and between defence and growth traits in white spruce

Defence traits	Piceol	Pungenol	*Pgβglu-1* transcripts	Average ring width	Total tree height	Stem diameter at breast height
Picein	0.38 (0.06) ^a^	−0.15 (0.07)	− 0.10 (0.06)	0.12 (0.07)	0.002 (0.06)	0.01 (0.06)
Piceol		0.64 (0.05)	0.43 (0.06)	0.05 (0.07)	0.06 (0.07)	0.10 (0.07)
Pungenol			0.57 (0.06)	−0.04 (0.07)	0.06 (0.07)	0.07 (0.07)
*Pgβglu-1* transcripts				−0.08 (0.06)	−0.01 (0.06)	− 0.04 (0.06)

^a^In parentheses, standard errors

**Table 6:** Factor loadings of the three first principal components (PC) for all defence and growth traits analysed in this study

Traits	PC1	PC2	PC3
Picein	−0.09	0.03	−0.86
Piceol	−0.28	0.51	−0.35
Pungenol	−0.23	0.55	0.26
*Pgβglu-1* transcripts	−0.16	0.51	0.18
Average ring width	−0.52	−0.22	0.12
Total tree height	−0.56	−0.20	0.10
Stem diameter at breast height	−0.49	−0.27	− 0.06

**Fig. 4:**
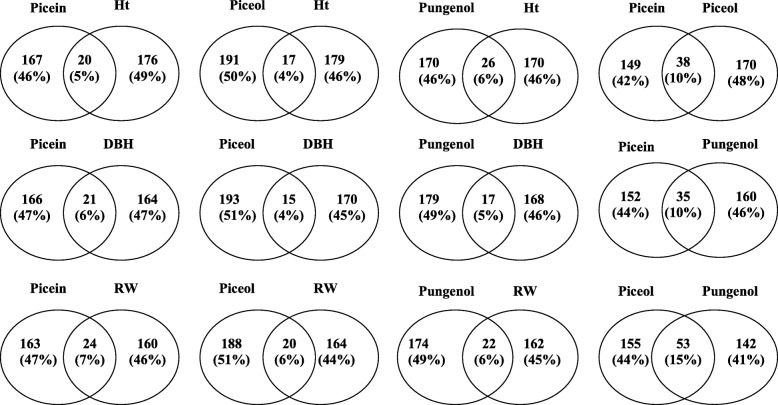
Venn diagrams indicating the extent of overlaps of significantly associated genes between defence traits and between defence and growth traits at *P* < 0.05**.** Abbreviations: Ht, total tree height; DBH, diameter at breast height; RW, average ring width

Comparisons of significantly associated genes for the different traits showed a small overlap between defence and growth traits (Fig. 4). Using a relaxed significance threshold of *P* < 0.05, the analyses identified close to 200 significant genes for the traits tested, and the proportion of shared significant genes ranged from 4% between piceol and stem diameter to 7% between picein and growth ring width (Fig. 4). In comparison, the overlap was two to three times higher among the defence traits and ranged from 10 to 15%. This low level of observed overlap is consistent with the weak phenotypic correlations that were observed between defence and growth traits (Table 5), thus suggesting little or no trade-off.

## Discussion

Despite the economic and ecological importance of white spruce and other conifers that are attacked by the spruce budworm in North American forests, very little is known of their naturally-occurring defence mechanisms. This study aimed to contribute to the understanding of the molecular basis of SBW defence traits described by [19, 29]. Previous work has linked SBW resistance to the foliar accumulation of the acetophenones piceol and pungenol [19] and *Pgβglu-1* gene transcripts [29] based on the analysis of 20 selected white spruce trees. The glycosylated acetophenone conjugates picein and pungenin were not linked to resistance although they accumulated to high levels in several trees [19, 29]. A recent study of full-sib families and clonal lines in white spruce found that these same chemical defences traits were moderately to highly heritable in field grown trees of six to 14 years of age [30]. Here, we looked at genetic variation and studied the molecular basis of these traits in a sample of 211 trees from as many open-pollinated families representing 42 natural populations, which were gathered and raised in a common garden experiment [32]. We identified 33 genes carrying a total of 35 SNPs significantly associated with one or more of the traits, and found that most of the genes were strongly expressed in the foliage. Acetophenones and their glycosylated conjugates accumulated to high levels in some individuals but no trade-offs were observed between defence and growth traits. We discuss the insights that are gained from these molecular analyses into the genetic control of SBW resistance.

### Candidate genes associated with defence traits

The association genetics results presented above support a few major findings. First, several of the significantly associated genes with known predicted functions were linked to defence or included genes that have been recently found to be indirectly implicated in the biosynthesis of phenolic compounds [71, 72]. Second, the multi-locus approach allowed to identify the largest number of significant SNPs that explained a larger proportion of the phenotypic variance. Here, SNPs identified with the MLMM approach explained 20% to 43% of the phenotypic variation. In contrast, the single-locus approach only identified two significant SNPs at most for each trait, and each SNP explained only a small proportion of the phenotypic variance, as observed in several other studies in forest trees [31–33, 73–75].

Robertson [76] proposed an exponential distribution model for quantitative traits in which there are few genes with large effects and many additional genes with small effects. The exponential model represents an alternative to the infinitesimal model where a large number of loci with individual small effects contribute to the quantitative genetic variation of the trait [73, 77, 78]. Our association genetics results suggest a genetic architecture that may be closer to the exponential model for acetophenone compounds and *Pgβglu-1* expression in white spruce trees. This interpretation is supported by MLMM results, which showed that a few significant genes collectively explain a large proportion of the phenotypic variation (e.g. 43% for piceol), suggesting a genetic architecture involving a moderate number of genes. It has been shown that traits involved in resistance to biotic stress may favour fixation of large-effect QTLs, and these QTLs are more common than predicted by the infinitesimal model of genetic adaptation [79]. This interpretation is also consistent with the report of moderate to high heritability for defence compounds against SBW in white spruce [30], and with other studies on secondary metabolites that contribute to biotic resistance in plants. Research on the variation in concentration of different terpenes also suggested that they are under the control of a few major genes in conifer trees [80], eucalyptus trees [81, 82], and crop plants [83, 84].

We also observed that a large proportion of the phenotypic variation for acetophenone metabolites remained unexplained in the present association genetics study [39]. This was expected, given that genotyping data were obtained for around 10% of the transcribed genes according to conservative estimates of the gene content for spruces [48, 85].

### Molecular basis of acetophenone accumulation

We observed that acetophenones and their glucoside conjugates reach high levels of accumulation. For instance, picein accumulated to 62.4 mg/g on a dry weight basis on average and reached much higher levels in some trees. The larvae of SBW feed primarily on newly formed foliage of spruce and fir trees in late Spring and early Summer [19, 39] and the temporal accumulation of acetophenones in the foliage is tightly linked to SBW resistance in white spruce [29, 39]. Acetophenones are thought to be derived from the phenylpropanoid pathway. However, most of the steps leading to their biosynthesis have only been proposed [86] and two genes have been shown to be directly involved in their accumulation and are involved in their glycosylation (*PgUGT5b*) [87] and deglycosylation (*Pgβglu-1*) [29]. The high levels of accumulation of acetophenones suggest that they represent a significant sink involving both phenolic and carbohydrate metabolisms. The predicted functions of the genes that we identified by association genetic approaches shed a first light onto the network of genes that may influence their synthesis and accumulation. The potential contribution of the genes is supported by data showing that nearly all of them are strongly expressed in white spruce foliage based on the transcript accumulation profiles of Raherison et al. [70]. In the following sections, we discuss our findings in light of the putative functions of the genes identified by genetic association analyses and of their potential involvement in plant metabolism.

### Phenolic metabolism

In this study, glutamine synthetase (GS) was associated with piceol and its glycosylated form picein with the MTMM approach. In conifers, GS has been shown to be responsible for the re-assimilation of ammonium provided by the deamination of phenylalanine, the precursor for phenylpropanoid biosynthesis, in the reaction catalysed by the enzyme phenylalanine ammonia-lyase (PAL) [88, 89]. This is an efficient nitrogen recycling system that was hypothesized to be responsible for the lack of trade-off between the accumulation of phenolic compounds and the growth of leaves or long shoots in birch [90]. Defoliation by herbivores alters the balance between nitrogen (N) sources and sinks [91] and to avoid severe N deficiency, plants have evolved an efficient N-recycling mechanism, which involves the GS enzyme system [92, 93].

Conifers produce diverse phenolic compounds that are involved in chemical defence against natural enemies [94]. The acetophenones picein and pungenin accumulate constitutively in white spruce and are believed to be synthesized via the phenylpropanoid pathway, which is also central to the synthesis of many chemical defences as well as lignin [19, 71]. However, much less is known about the genes that may be involved in the molecular regulation of the acetophenone specific branch [86]. The genes putatively involved in the phenylpropanoid pathway have been characterized in white spruce [28] and many of them were up-regulated in response to fungal infection or herbivory attack, suggesting a role in conifer defence [3]. Here, none of the core phenylpropanoid pathway genes were significantly associated genetically with the accumulation of acetophenones, although they were represented on the genotyping array. This may be explained based on two major considerations. First, most of the phenylpropanoid pathway genes are part of superfamilies in spruce [3, 28, 71] and those which are key to foliar defence may not have been adequately represented on the genotyping chip and thus may have been untested (Additional file 1: Figure S1). For example, 37 OMT /COMTL genes were identified in white spruce [71] and several of them were differentially expressed between tissues and in response to stress factors [3]. Secondly, most of the phenylpropanoid genes tested had low expression levels in foliage tissue (Additional file 1: Figure S1). This pattern suggests that other phenylpropanoid genes should be tested and selected based on the recently developed understanding of gene families [28] and expanded expression data in white spruce [95].

### Carbohydrate metabolism

In this study, two UDP-D-Glucuronate 4-epimerases (GAEs) (GAE1 and GAE4) were significantly associated with acetophenones and *Pgβglu-1* expression. GAE1 was associated with picein by the SLMM and MLMM approaches and both picein and pungenol by the MTMM approach; GAE4 was only associated with *Pgβglu-1* transcript levels. Glycosylation consists of the attachment of a sugar moiety to phenolic compounds and is important to enhance their stability and solubility and reduce their toxicity [96]. Glycosylation by glycosyltransferases (GT) involves the transfer of sugar from its activated nucleotide sugar donor to specific acceptor molecules. One of the common glycosyl donors in plants is UDP-glucuronate [97]. UDP-glucuronic acid (UDP-GlcA) is made from UDP-Glc via the UDP-Glc dehydrogenase activity [97]. UDP-D-Glucuronate 4-epimerases (GAEs) catalyse the reversible interconversion of UDP-D-GlcA and UDP-D-GalA [98]. One other GT gene (O-fucosyltransferase family protein) was also associated with pungenol. Plant O-fucosyltransferases (O-FuTs) are a type of GTs that catalyses the transfer of the nucleotide sugar fucose from the donor, guanosine diphosphate fucose (GDP-Fuc), to various acceptor molecules. Taken together, these observations indicate that GT enzymes influence the accumulation of at least one of the acetophenone glucoside and one of the aglycons. Functional experimentation could establish whether any of these sequences may act directly on piceol and pungenol to form the corresponding glucosides.

### Oxidative stress control

Several of the genes identified here by association testing were potentially involved in detoxifying systems to protect cells from oxidative damage. The physiological link between acetophenones or the level of *Pgβglu-1* gene transcripts on one hand, and the oxidative stress related genes on the other hand, is unknown. But such a link is suggested by indications that piceol is cytotoxic for plant cells [99] in addition to some insects [19] and fungal pathogens [100].

The genes identified here by association genetics testing included an ascorbate peroxidase (APX) enzyme, which controls the hydrogen peroxide (H_2_O_2_) concentration in cells by catalysing its conversion to water using ascorbate as an electron donor [101, 102]. An increase in the cellular H_2_O_2_ concentration in *Arabidopsis thaliana* is known to trigger DNA damage [103]. The genes SOG1, a NAC transcription factor that regulates DNA damage response [104, 105] and CYCP4;1, a cyclin, are reported to prevent oxidative damage and were both associated with pungenol and piceol. The SOG1 transcription factor regulates cyclin-dependent kinases (CDK) inhibitor genes *SMR4*, *SMR5*, and *SMR7* (belonging to the *SIAMESE/SIAMESE-RELATED* class), which are transcriptionally activated by DNA damage [103]. Cyclins are regulatory proteins that interact with CDKs to control progression through the cell cycle [106].

One of the genes significantly associated with *Pgβglu-1* transcripts encoded a phenylcoumaran benzylic ether reductase (*PCBER1*) [71], which has been reported to participate in the biosynthesis of important plant defence compounds [71, 107, 108]. Niculaes et al. [72] showed that *PCBER1* may protect against oxidative damage by chemically reducing phenylpropanoid dimers in poplar xylem. Another gene associated with *Pgβglu-1* transcripts encoded a glutathione S-transferases (GST), which is involved in detoxification in plants [109]. In poplar trees, expression of GST increased in leaves following herbivory by forest tent caterpillars [3].

### Trade-offs between defence and growth traits

We considered both the phenotypic correlations between traits and the list of genes significantly associated with the various traits analysed, and found little evidence for trade-offs between defence traits against SBW and growth in white spruce. These results suggest that the cost of constitutive production of piceol and pungenol as secondary metabolites does not affect primary metabolism needed to sustain growth, which is consistent with previous results in other forest trees. Similar findings were reported in a previous investigation on white spruce resistance to SBW based on the analysis of full-sib families and clonal lines where low and non-significant genetic correlations were observed between defence and growth traits [30]. In Douglas-fir and Scots pine, the accumulation of constitutive phenolic compounds in bark was also not correlated with growth [110, 111]. In terms of biosynthetic costs, phenolic compounds have also been suggested to be less costly to produce than other compounds such as alkaloids, which have a higher energy requirement to make inorganic nitrogen bioavailable (reviewed in [112]).

Among the three acetophenones studied here, the glycosylated acetophenone picein was the most abundant, and by far, as it made up 6% of the total needle dry mass on average. High foliar concentrations of phenolic compounds were also observed in other tree species and were correlated with resistance to herbivores. For instance, condensed tannins represented over 10% of the dry mass in birch leaves [90, 113] and phenolic glycosides constituted up to 4% of leaf dry weight in aspen (*Populus tremuloides*) [114]. Picein production may function as a reservoir for storing sugar (carbohydrates) in white spruce foliage. In silver birch, it has been shown that some phenolic compounds may act as a reservoir for the synthesis of other phenolic compounds when the phenylpropanoid metabolism is activated, and storing surplus carbon as cinnamoylquinates would be a better defence against herbivory than the accumulation of storage carbohydrates such as starch, thus potentially allowing a more rapid response to environmental threats [115, 116].

## Conclusions

The present study represents a first step in understanding and dissecting the genetic architecture of defence traits against SBW in white spruce. We explored three different association genetics testing approaches and, taking advantage of the genomic resources developed for white spruce, we detected 33 genes carrying SNPs significantly involved in the observed variation for defence traits. Our results indicate that the multi-locus association genetic approach is more powerful than the single-locus approach for identifying candidate genes implicated in the constitutive defence against SBW. We further showed that these traits are likely to be under the mixed control of minor and major genes with no significant trade-offs with growth traits. The present results should open up new opportunities for functional studies to determine the molecular roles of these genes in influencing SBW resistance. In addition, these genes and a more complete determination of their polymorphisms should allow to develop molecular tools to help identify and breed trees that are more resistant to SBW, which have been lacking to date. These tools may thus represent a means to shorten the long periods of time that tree breeders need to assess defence against SBW in the field.

## Additional file

Additional file 1: **Figure S1.** Heatmap of tissue-specific expression pattern of candidate genes involved in phenylpropanoid pathway used in this study and their functional annotations. Expression data are from the PiceaGenExpress database [70]. Columns represent vegetative tissues: F, foliage; B, vegetative buds; XM, xylem–mature; XJ, xylem–juvenile; P, phelloderm; R, adventitious roots; M, megagametophytes; E, embryogenic cells; transcript levels represent relative abundance classes within each tissue, grey is for missing data; ND, not detected. (DOCX 128 kb)

## Abbreviations

DBH: Stem diameter at breast height
FDR: False discovery rate
GO: Gene ontology
GWAS: Genome-wide association studies
Ht: Tree height
LD: Linkage disequilibrium
MLMM: Multi-locus mixed model
MTMM: Multi-trait mixed model
PCA: Principal component analysis
Pgβglu-1: *Picea glauca* β-glucosidase-1
PVE: Phenotypic variation explained
QTL: Quantitative trait locus
RW: Ring width
SBW: Spruce budworm
SLMM: Single-locus mixed model
SNP: Single-nucleotide polymorphism
