# Supplementary material for: Association genetics of acetophenone defence against spruce budworm in mature white spruce
Source: BMC Plant Biol. 2018 Oct 12;18:231. doi: 10.1186/s12870-018-1434-y (PMC6182838; doi:10.1186/s12870-018-1434-y)
Supplement: Supplementary file 1 — Figure S1. Heatmap of tissue-specific expression pattern of candidate genes involved in phenylpropanoid pathway used in this study and their functional annotations. Expression data are from the PiceaGenExpress database [70]. Columns represent vegetative tissues: F, foliage; B, vegetative buds; XM, xylem–mature; XJ, xylem–juvenile; P, phelloderm; R, adventitious roots; M, megagametophytes; E, embryogenic cells; transcript levels represent relative abundance classes within each tissue, grey is for missing data; ND, not detected. (DOCX 128 kb) [file 12870_2018_1434_MOESM1_ESM.docx]

| **Enzymes** | **GenBank** | **Functional annotation** | **F** | **B** | **XM** | **XJ** | **P** | **R** | **M** | **E** |  |  |  |
| --- | --- | --- | --- | --- | --- | --- | --- | --- | --- | --- | --- | --- | --- |
| **Phenylalanine Ammonia-Lyase (PAL)** | BT112211 | PAL1 |  |  |  |  |  |  |  |  |  |  |  |
|  | BT119163 | PAL2 |  |  |  |  |  |  |  |  |  |  |  |
|  | BT114680 | PAL4 |  |  |  |  |  |  |  |  |  |  | ND |
| **Caffeoyl-Coenzyme A O-Methyltransferase (CCoAOMT)** | BT116840 | CCoAOMT |  |  |  |  |  |  |  |  |  |  | low |
|  | BT106698 | CCoAOMT |  |  |  |  |  |  |  |  |  |  | Transcript level |
|  | BT106398 | CCoAOMT |  |  |  |  |  |  |  |  |  |  |  |
|  | BT109398 | CCoAOMT |  |  |  |  |  |  |  |  |  |  |  |
| **Cinnamoyl Coa Reductase (CCR)** | BT110936 | CCR1 |  |  |  |  |  |  |  |  |  |  |  |
|  | BT113236 | CCR1 |  |  |  |  |  |  |  |  |  |  |  |
|  | BT111802 | CCR1 |  |  |  |  |  |  |  |  |  |  |  |
| **Cinnamyl Alcohol Dehydrogenase (CAD)** | BT112280 | CAD5 |  |  |  |  |  |  |  |  |  |  |  |
|  | BT103167 | CAD9 |  |  |  |  |  |  |  |  |  |  |  |
|  | BT119677 | CAD6 |  |  |  |  |  |  |  |  |  |  | high |
|  | BT116920 | CAD |  |  |  |  |  |  |  |  |  |  |  |
|  | BT115412 | CAD |  |  |  |  |  |  |  |  |  |  |  |
| **4-Coumarate:CoA Ligase (4CL)** | BT116171 | 4CL3 |  |  |  |  |  |  |  |  |  |  |  |
|  | DR551141 | 4CL2 |  |  |  |  |  |  |  |  |  |  |  |
|  | BT106671 | 4CL2 |  |  |  |  |  |  |  |  |  |  |  |
| **p-Coumarate 3-Hydroxylase (C3H)** | BT106474 | C3H |  |  |  |  |  |  |  |  |  |  |  |
|  | BT116913 | C3H |  |  |  |  |  |  |  |  |  |  |  |
| **Caffeic acid O-Methyltransferase (COMT)** | BT108042 | COMT-1 |  |  |  |  |  |  |  |  |  |  |  |
|  | BT117092 | COMT-1 |  |  |  |  |  |  |  |  |  |  |  |
|  | BT102643 | COMT-1 |  |  |  |  |  |  |  |  |  |  |  |
|  | BT107167 | COMT-1 |  |  |  |  |  |  |  |  |  |  |  |
| **Hydroxycinnamoyl Transferase (HCT)** | BT117023 | HCT |  |  |  |  |  |  |  |  |  |  |  |
|  | BT111180 | HCT |  |  |  |  |  |  |  |  |  |  |  |
|  | BT101292 | HCT |  |  |  |  |  |  |  |  |  |  |  |
|  | BT118162 | HCT |  |  |  |  |  |  |  |  |  |  |  |
| **Ferulic acid 5-Hydroxylase 1 (F5H)** | BT117620 | F5H1 |  |  |  |  |  |  |  |  |  |  |  |
